# Assessment of students’ attitude and level of community involvement in community-based education at training sites in Gedeo zone, South Ethiopia

**DOI:** 10.1186/s13104-018-3940-2

**Published:** 2018-11-26

**Authors:** Mohammed Feyisso Shaka, Gedefa Amenu Senbeto

**Affiliations:** 1 School of Public Health, Dilla University College of Health Sciences and Medicine, Dilla, Ethiopia; 2 Department of Midwifery, Dilla University College of Health Sciences and Medicine, Dilla, Ethiopia

**Keywords:** Community-based education, Students’ attitude, Community involvement

## Abstract

**Objective:**

This study was aimed to assess the students’ attitude towards community based education and the level of community involvement in community-based education.

**Result:**

Among the 634 community members participated in this study, 64.4% were aware of the community-based education program and 86.5% of these participants have favorable attitude towards the program. The level of community involvement in the activities of students in this study is 40.9%. About 72% of the students have favorable attitude towards community based education. Female students were more likely to have favorable attitude toward the program [AOR: 7.64 (CI 1.80, 32.50)]. On the other hand, students who have low participation during group assignment [AOR: 0.07 (CI 0.01, 0.95)] and those with lower socially conscious attitude [AOR: 0.02 (0.01, 0.08)] were less likely to have favorable attitude.

**Electronic supplementary material:**

The online version of this article (10.1186/s13104-018-3940-2) contains supplementary material, which is available to authorized users.

## Introduction

Innovative medical schools currently use community-based education (CBE) as one of the means for training of health experts who are responsive to the needs of a society [1]. During CBE, community is used extensively as a learning environment. Furthermore, there is active engagement of students, instructors, community members, different sectors and agencies throughout the community based-education process. CBE is commonly operated by training institutions to enhance the quality of education, and also as a means to retain health professionals in underserved areas. This approach was primarily arose to contribute for “Health for All” strategy through enhancing PHC program [2].

CBE is aimed to widen students’ insight of health problems of a community through their learning, service and research work [3]. It involves identifying of community health problems (community diagnosis) and searching for solutions through engagement of stakeholders in the community [4–6].

The two commonly practiced community-based education programs in medical trainings in Ethiopia are community based training program (CBTP) and team training program (TTP) [7]. The practice of each community-based education varies from country to country and from institution to institution. The students’ learning process is spiral in nature and problem-solving in approach. During their stay in the community, students exercise different learning oriented community based activities including data gathering, community diagnosis, planning, implementation and evaluation. During their active involvement in this program, the students gain a sense of social responsibility and detail understanding of the community health and social problems as well as solutions within local resources [4, 5, 8, 9].

CBE is a powerful means of improving quality of the community health services. Students also acquire a skill to mobilize the community in implementing community development projects and using available community resources. The community resources could be in the form of labor, local material, space or money (in cash or in kind) to cover most of the intervention costs.

The main focus of this study was to assess the attitude of students toward CBE and the level of community involvement on activities during the program.

## Main text

### Methods

#### Study design and area

The institution and community-based quantitative study, which was supplemented by a qualitative enquiry, was conducted from March 6, 2017 to June 28, 2017. The study population was comprised of the community members of three towns (Dilla, Yirgacheffie, and Wonago) in the Gedeo zone of Ethiopia and final-year students from all the departments of the Dilla University College of Health Sciences and Medicine who had community-based education (CBE) as part of the core competency in their education curriculum [10].

#### Sample size calculation and sampling technique

The sample size of students was the entire graduating class of students from the specified departments. For community-based, the sample size was calculated using a single population proportion formula as follows:

$${\text{N}}\, = \,\frac{{{\text{Z}}\frac{\upalpha}{2}{\text{pq}} }}{{{\text{d}}2}},$$

where $${\text{Z}}\frac{\upalpha}{2}$$ = 1.96, p = 50%, q = 1−p = 50%, and d = 0.5.

The calculated sample size was 384, after considering the design effect of 1.5 and a 10% nonresponse rate, the final sample size was 634. The sample size for the qualitative study was determined based on the data saturation.

Three kebeles (local districts) from Dilla, one from Wonago, and two from Yirgacheffie were selected using a simple random sampling method. The respondents were selected by a systematic random sampling method based on the arrangement of the households in the selected towns.

#### Data collection instruments and personnel

The tools used in the study were developed by reviewing the literature that was pertinent to the study. Cronbach’s alpha reliability coefficient was checked for each question, resulting in ≥ 0.7. For the data collection among students, three facilitators were recruited to assist in the completion of the questionnaire. Eleven data collectors and three supervisors were used for the community-based house-to-house data collection.

#### Measurement

The attitude of the students toward CBE was assessed by using a range of questions that were measured with a Likert scale. Scores greater than the average value were classified as having a favorable attitude. To create the scale for the socially-conscious attitude of the students, two asked the reason why they selected health science as their future career. The statements were “Helping people to improve their health” and “Having a role in improving community health.” Scores ranged from 2 to 10, and scores from 2 to 6 were categorized as low, scores from 7 to 8 were categorized as average, and scores of 9 to 10 were categorized as high. The level of the community members’ involvement was also assessed by using questions that inquired about participation in the activities of students during the CBE.

#### Data analysis

Data was entered into a template format prepared from EpiData version 3.1 and exported to SPSS version 20 for analysis. Descriptive statistics were computed for the attitude of students toward CBE and the level of involvement of the community. Logistic regression was used to assess factors associated with attitude of students towards CBE.

### Result

#### Awareness and attitude of communities and students towards community-based education

Out of the 634 community members expected to participate in the study 631 successfully responded. Regarding the awareness of community members, 406 (64.4%) were aware of the presence of the CBE program in their locality. Among them, 347 (86.5%) have a favorable outlook towards students’ activities during community-based education (Table 1). However, only 146 (36.6%) of the respondents rated good or above on the scale for the contribution of community-based education program to improve the health of the community in the study area, whereas 166 (41.6%) rated fair and 87 (21.8%) rated poor or below.

**Table 1:** Awareness and attitude of communities about the community-based training program and their involvement in CBE activities, Gedeo zone, South Ethiopia, July 2017

No.	Variables	Frequency	Percentage (%)
1.	Heard or seen CBE activities of students	630	100%
	Yes	406	64.4
	No	224	35.6
2.	Attitude of community toward CBE		
	Favorable	347	86.5
	Neutral	47	11.7
	Unfavorable	7	1.7
3.	Participated on the students activities	401	100%
	Yes	164	40.9
	No	237	59.1
4.	Way of involvement in students’ activities	167	100%
	Participating personally on activities	120	73.1
	Providing money/material	3	1.8
	Mobilizing communities	44	26.8
5.	Reported reason for not participating in CBE activities	235	100%
	Not around	84	35.4
	No invitation from students	122	51.5
	Don’t want to participate	8	3.4
	No skill to participate	7	3.0
	Other reason	14	5.9

Out of the 197 graduating class students of June 2017 in Dilla University College of Health Sciences and Medicine, 188 participated in this study. Based on a measurement of the socially conscious attitude of the students, 129 (70.5%) were enthusiastic towards helping people, i.e., they have average or above socially conscious attitude. In comparison, about 133 (72%) of the students have a favorable attitude towards community-based education.

Regarding the perceived benefit of community-based education for students and community, only 56 (30.1%) of the students reported that they have gained high competence from CBE. The overall students’ evaluation of their skills obtained from CBE is a little above average (3.6 out of 6). However, about 83.2% of the students rated the advantage of CBE to community as average or above (Table 2; Additional file 1).

**Table 2:** Students rating of community involvement in CBE practice and importance of CBE, Gedeo Zone, South Ethiopia, July 2017

No.	Variables	Frequency	Percentage (%)
1.	Community involvement	188	100%
	Poor	48	25.5
	Average	51	27.1
	High	89	47.3
2.	Support from health sectors in the area	188	100%
	Poor	37	20.3
	Average	65	35.7
	High	80	44.0
3.	Advantage of CBE for community	184	100%
	Poor	31	16.8
	Average	73	39.7
	High	80	43.5
4.	Perceived competence obtained from CBE	182	100%
	Low	36	19.7
	Fair	91	49.7
	High	56	30.6
	Average	3.6	–

#### Participation of communities in the activities of students during CBE practice

Among the 402 community members who were aware of the presence of the CBE program in their locality, only 164 (40.9%) had ever participated in CBE activities (Table 3). Most of these participants were involved during the initial stage of data collection (43.3%) and during the implementation/intervention phase (45.7%). The level of participation during prioritization and planning was significantly limited, which is 8% and 6% respectively. Similarly, < 50% of the students rated the overall community participation in CBE to be high, whereas only 44% rated above average for support from health sector institutions (Table 2).

**Table 3:** Factors associated with students’ attitude towards community-based education among students of College of Health Sciences and Medicine, Dilla University, South Ethiopia, July 2017

No	Variables	Attitude		COR (95% CI)	AOR (95% CI)
		Not favorable n = 51 (27.7%)	Favorable, n = 133 (72%)		
1.	Age category				
	22 and below	18	57	1	1
	23 and above	33	76	0.73 (0.37, 1.42)	1.77 (0.53, 5.95)
2.	Sex				
	Male	31	78	1	1
	Female	20	55	1.09 (0.56, 2.11)	7.64 (1.80, 32.50)
3.	Department				
	Health officer	20	81	1	1
	Midwifery	7	13	0.46 (0.16, 1.30)	5.18 (0.94, 28.60)
	Psychiatry	17	5	0.07 (0.02, 0.22)	0.46 (0.07, 3.11)
	Medicine	7	34	1.20 (0.46, 3.10)	2.15 (0.44, 10.50)
4.	Section				
	Regular	47	98	1	1
	Extension	4	35	4.20 (1.41, 12.50)	0.32 (0.07, 1.55)
5.	Participation during group work				
	Low	11	2	0.05 (0.01, 0.22)	0.07 (0.01, 0.95)
	Fair	23	59	0.63 (0.31, 1.29)	0.23 (0.07, 0.79)
	High	17	69	1	1
6.	Joined the department by choice				
	Yes	33	113	1	1
	No	18	20	0.32 (0.15, 0.68)	2.67 (0.51, 14.26)
7.	Satisfaction with the joined department				
	Not satisfied	7	15	0.48 (0.17, 1.31)	0.31 (0.08, 1.23)
	Neutral	22	18	0.18 (0.08, 0.39)	0.27 (0.06, 1.30)
	Satisfied	22	99	1	1
8.	Socially conscious attitude				
	Low	37	16	0.04 (0.02, 0.11)	0.02 (0.01, 0.08)
	Average	6	30	0.49 (0.16, 1.54)	0.96 (0.26, 3.62)
	High	8	81	1	1

#### Factors associated with students’ attitude toward CBE

In this study attempts were made to assess factors associated with the students’ attitude towards community-based education. As it can be noted from the result, sex of the students, perceived level of the students’ participation during usual group assignments in the class and their socially conscious attitude were associated with the attitude towards community-based education.

Accordingly, female students were nearly eight times more likely to have favorable attitude towards community-based education compared to male students [AOR: 7.64 (CI 1.80, 32.50)]. On the other hand, students whose participation during usual group assignment in a class was reported to be low were 93% less likely to have favorable attitude toward CBE compared to those who have higher participation [AOR: 0.07 (CI 0.01, 0.95)]. Similarly, the likelihood of having favorable attitudinal outlook towards CBE was decreased by 98% for students whose socially conscious attitude was low compared to those with higher socially conscious attitude [AOR: 0.02 (0.01, 0.08)] (Table 3).

### Discussion

In this study, most community members and stakeholders had a favorable attitude toward the activities performed by students during CBE. This finding is consistent with the findings from other studies [7, 11]. This is also supported by the findings from a qualitative study. A health extension worker from one of the towns said, “The students can significantly contribute to the improvement of the health of the communities and support our routine work” and the head of a certain health office stated, “The students can contribute much to improve the health of the community from what they have learned….”

Approximately 72% of the students indicated a favorable attitude towards CBE, which also coincided with the socially conscious attitude of students that was used to measure their enthusiasm. This is supported by the findings from a study conducted in Iran [11]. Female students were found to be more likely to have a favorable attitude toward CBE. This may have been due to the difference in the behaviors of males and females. It also demonstrated that students who are more enthusiastic about serving the community have a more favorable attitude toward CBE since the pillar of CBE activities is understanding the specific problems of the communities and developing strategies to tackle the problems. Students who had a poor rating on their participation level for typical group assignments during class-based/clinical attachments were less likely to have a positive attitude toward CBE. This is due to the fact that students who had participated in previous group assignments had a good exposure to the teamwork that they would actually face during CBE.

Regarding the level of involvement of the communities in the activities of students during CBE, only 40.9% participated in the students’ activities. In addition, the extent of participation was relatively low for those communities that did not participate during key periods of the program, such as prioritization and planning. Since communities are the main stakeholders of the CBE practice, this level of involvement is much lower than it should be and may be related to the inadequate effort of students to mobilize and involve community members. Students were rated as less than average for their efforts in communicating about each CBE activity within the community. This is similar to the findings from the study conducted at Jimma University where the overall community participation in CBE was found to be low [7, 12].

According to the results of the qualitative section of this study, the level of involvement of the people responsible, including health extension workers, is relatively poor. For example, a 32-year-old health extension worker from one of the towns stated, “I haven’t participated in any of the students’ activities, I haven’t asked them about what they do, and they don’t communicate anything about their activities with us.” Although participation of the health center in the study area is relatively better, it is substantially limited. There is limited communication between the heads of the health office or health center and the students when preparing an action plan and its implementation, as well as poor communication of details and intervention reports with the stakeholders. The head of a certain health center in the study area stated that “one problem I realized through my work experience here is that they don’t consult us during the planning and implementation. They also do not communicate with us to let us know what they found in the community and what they improved.” The findings specifically suggest that the students’ and the supervisors’ behaviors for motivating and mobilizing communities to participate in CBE activities is poor.

In general, the level of community involvement is substantially low and the students’ attitude toward CBE is not adequate. Since communities are the main stakeholders of CBE, a special arrangement to tangibly improve community involvement in the program and develop a sense of ownership needs to be created.

## Limitation of this study

Scope of the study being limited to one cohort of medical school students.

## Additional file

**Additional file 1.** Socio-demographic characteristics of students and community members.

## Abbreviations

CBE: community based education
CBTP: community based training program
HEW: health extension workers
PHC: primary health care
SPSS: statistical package for social science
TTP: team training program

## References

[1] Talib ZM, Baingana RK, Sagay AS, Van Schalkwyk SC, Mehtsun S, Kiguli-Malwadde E (2014). Investing in community-based education to improve the quality, quantity, and retention of physicians in three African countries. HHS Public Access.

[2] Woldemariam T, Asefa M (1990). Historical perspective of Jimma Institute of Health Sciences. Bull JIHS.

[3] Diab P, Flack P (2013). Benefits of community-based education to the community in South African health science facilities. Afr J Prim Health Care Fam Med.

[4] UNESCO. International conference on education, 38th session, Geneva, 10–19 November 1981. In Paris; 1982. p. 38.

[5] WHO. Community based education for health personnel. Technical report series 746. Geneva: WHO; 1987. 3632772

[6] Richards R, Fulop T. Innovative schools for health personnel. WHO offset Publication, No. 102. Geneva; 2007. 3424994

[7] Tegegne M, Asefa M, Tessema F, Kebede K (2000). Assessment of the community-based training programme at Jimma University, Ethiopia. Ethiop J Health Dev.

[8] Asefa M (2000). Community-based education: concept and practice. Ethiop J Health Dev..

[9] Richards R, Fulop T. Innovative schools for health personnel. WHO offset Publication, No. 102. Geneva; 1987. 3424994

[10] Dilla University (2017). Dilla University community based education guideline.

[11] Movahhed T, Ajami B, Ghasemi H, Shakeri T, Sufiani M, Health CO (2013). The attitude toward community based education among dental students at an Iranian Dental School. Future Med Educ J.

[12] Ibrahim N, Bekele M (2014). Assessing the impact of community based training program of Jimma University on improving livestock health in Jimma Zone. Afr J Basic Appl Sci.

