# Supplementary material for: Assessment of students’ attitude and level of community involvement in community-based education at training sites in Gedeo zone, South Ethiopia
Source: BMC Res Notes. 2018 Nov 26;11:835. doi: 10.1186/s13104-018-3940-2 (PMC6258147; doi:10.1186/s13104-018-3940-2)
Supplement: Supplementary file 1 — Additional file 1. Socio-demographic characteristics of students and community members. [file 13104_2018_3940_MOESM1_ESM.docx]

**Additional Tables**

Socio-demographic characteristics of the community respondents in Gedeo zone, South Ethiopia, July 2017

| **No.** | **Variables** | **Frequency** | **Percentage (%)** |
| --- | --- | --- | --- |
|  | Age category | 631 |  |
|  | 30 years or less | 229 | 36.3 |
|  | 31 to 40 | 191 | 30.3 |
|  | Above 40 | 211 | 33.4 |
|  | Occupation | 606 |  |
|  | Merchant | 174 | 28.7 |
|  | government employee | 159 | 26.2 |
|  | Housewife | 162 | 26.7 |
|  | Private Employee | 54 | 8.9 |
|  | Other | 57 | 9.4 |
|  | Educational Level | 627 | 100% |
|  | No formal education | 88 | 14.0 |
|  | Primary education | 178 | 28.4 |
|  | Secondary education | 214 | 34.1 |
|  | College and above | 147 | 23.4 |
|  | Any position in the community | 610 |  |
|  | Yes | 39 | 6.4 |
|  | No | 571 | 93.6 |
|  | Level of participation on community events | 626 | 100% |
|  | Poor | 61 | 9.7 |
|  | Fair | 400 | 63.9 |
|  | Good | 165 | 26.4 |

Socio-demographic and academic characteristics of Students in Dilla University College of Health Sciences and Medicine, Dilla Ethiopia, July 2017

| **No.** | **Variables** | **Frequency** | **Percentage (%)** |
| --- | --- | --- | --- |
|  | Age category | 188 | 100% |
|  | 22 and below | 75 | 39.9 |
|  | 23 and above | 109 | 59.1 |
|  | Median | 23.00 | - |
|  | Section | 188 | 100% |
|  | Regular | 149 | 74.5 |
|  | Extension | 39 | 25.5 |
|  | Participation during group works | 185 | 100% |
|  | Low | 13 | 7.0 |
|  | Fair | 82 | 44.3 |
|  | High | 90 | 48.6 |
|  | Repeated class | 185 | 100% |
|  | Yes | 23 | 12.4 |
|  | No | 162 | 87.6 |
|  | Joined department by choice | 188 | 100% |
|  | Yes | 148 | 78.7 |
|  | No | 40 | 21.3 |
|  | Satisfaction level with the joined department | 187 | 100% |
|  | Very unsatisfied | 22 | 11.8 |
|  | Neutral | 42 | 22.5 |
|  | Satisfied | 123 | 65.7 |
